# The effects of intravenous, glucose versus saline on ovarian follicles and their levels of some mediators of insulin signalling

**DOI:** 10.1186/1477-7827-13-6

**Published:** 2015-01-20

**Authors:** Rex John Scaramuzzi, Nesrine Zouaïdi, Jean-Baptiste Menassol, Joëlle Dupont

**Affiliations:** L’Institut National de la Recherche Agronomique, Unité Mixte de Recherche 6175, Physiologie de la Reproduction et des Comportements, Nouzilly, 37380 France; Department of Comparative Biomedical Sciences, The Royal Veterinary College, Hawkshead Lane, North Mimms, Hertfordshire, AL9 7TA UK; L’Institut National de la Recherche Agronomique, UR 1213 URH Unité de Recherches sur les Herbivores, Centre de recherche de Clermont-Ferrand-Theix, Clermont-Ferrand, France

**Keywords:** Sheep, Ewe, Nutrition, FSH, Oestradiol, Aromatase, AMPK, Akt, ERK

## Abstract

**Background:**

A short-term increase in food intake and specifically dietary energy can stimulate folliculogenesis and increase ovulation rate in ewes. The mechanism appears to involve the insulin-glucose metabolic system and its interaction with FSH signalling pathways in the granulosa cells of ovarian follicles. This experiment was designed to investigate the interaction between these two systems in the granulosa cells of ovarian follicles.

**Methods:**

Thirty six Ile-de-France ewes were used in this controlled experiment to study the effects of intravenous glucose on folliculogenesis. Eighteen ewes were infused with glucose (10 mM/h for 72 h) from day 8 of the oestrous cycle, while the others (controls) received saline. Ovaries were collected when the infusions ended (luteal phase) or 30 h later and after a luteolytic dose of a PGF_2α_ analogue (follicular phase). Follicles were dissected and granulosa cells and follicular fluid harvested. The blood concentrations of glucose, insulin, oestradiol and FSH were monitored over the experiment. The levels of Aromatase P_450_ and of the phosphorylated and non-phosphorylated forms of Akt, AMPK and ERK in granulosa cells and the concentration of oestradiol in follicular fluid, were determined.

**Results:**

Glucose increased the circulating concentration of glucose (P < 0.05) and insulin (P < 0.05). It also increased the total number of follicles >1.0 mm in diameter (P < 0.05) and small (P < 0.05) follicles (>1.0 to 2.0 mm in diameter) but not medium (>2.0 to 3.5 mm in diameter) or large (>3.5 mm in diameter) follicles. Glucose decreased circulating oestradiol (P < 0.05) but not that of FSH or progesterone. Glucose reduced aromatase P_450_ (P < 0.05) and decreased the phosphorylation of Akt (P < 0.05), ERK (P < 0.05) and AMPK (P < 0.05) in granulosa cells from oestrogenic follicles. The level of Aromatase P_450_ was greatest in large oestrogenic follicles and the phosphorylation of Akt (P < 0.05), ERK (P < 0.05) and AMPK (P < 0.05) was lower in small follicles compared to medium and large follicles.

**Conclusions:**

These data suggest that the effect of glucose in small follicles is a direct action of glucose that increases the number of small follicles while the effect of glucose in oestrogenic follicles is an indirect insulin-mediated action.

## Background

Reproduction is subject to the influence of several factors related to the animal itself and to its environment. Among these, nutrition is one of the main factors affecting most aspects of the reproductive performance of the animal [1, 2]. Lindsay and his colleagues [1, 2] recognized that an increase in short-term food intake increased the lambing rate in ewes by stimulating ovulation rate. Three effects of nutrition on ovulation rate, the "acute", "dynamic" and "static" effects, have been described [2, 3] although there is uncertainty over the number of mechanisms involved [3]. The acute effect is an effect of diet associated with weight change, the static effect is associated with the absolute level of body weight while the dynamic effect is a short-term dietary effect not accompanied by a change in weight [reviewed in: 2, 3].

There have been numerous investigations of the relationship between diet and ovulation rate in farm animals and particularly sheep [2–4] and of the physiological mechanism(s) responsible for this phenomenon [3, 5, 6]. The inclusion of energy-dense foods such as lupin grain [7–13] or corn with soy meal [14] in the diet will increase both ovulation rate and the number of follicles. Other forms of nutritional manipulation for example, the infusion of leptin [15] or glucose [16–18] have been investigated and shown to increase both the ovulation rate and the number of follicles. So clearly, there is a link between nutrition and folliculogenesis. This relationship could involve effects mediated by the intrafollicular glucose-insulin system [3, 5, 6] and other energy sensing mechanisms such as the AMPK system [6, 19]. Although there is an extensive literature (see references cited above) describing the effects of dietary energy on folliculogenesis and ovulation rate there are relatively few studies of the intrafollicular mechanisms that are affected by the energy content of the diet. The level of Aromatase P_450_ was reduced in follicles of ewes whose diet was supplemented with lupin grain [20] or infused with glucose [18] and in the former study these effects were associated with alterations in the level of expression of the insulin receptor substrate (IRSs) proteins and in the later study, they were associated with alterations in the levels of Akt and AMPK; and Akt is a phosphorylation target in the insulin signalling pathway. In addition the presence of the insulin dependent glucose transporter (GLUT4 or the sugar transport facilitator [SLC2A-4]) has been confirmed in ovine [21], bovine [22] and rodent [23] ovarian follicles. Finally, insulin has been identified as a factor in several malfunctions of the ovary and its follicles in a number of ovarian pathologies [24]. These data suggest that insulin signalling to granulosa cells is implicated in the effects of dietary energy on folliculogenesis.

To improve the understanding of the interaction between folliculogenesis and the metabolic status of ewes we studied the effect of intravenous glucose on the insulin-signaling pathways in granulosa cells of follicles collected from ewes in either the luteal or follicular phases of the oestrous cycle. Although several potential metabolic sensors have been identified, including the IGF system, GH, and leptin [25, 26] we have focused our attention on the insulin-glucose and AMPK systems. A preliminary report of certain aspects of this study has been published [27].

## Methods

### Animals

The experiment was conducted at the INRA laboratory at Nouzilly in France between August and September using 36 Ile-de-France ewes. During the experiment the ewes were housed in group pens until they were fitted bilaterally with catheters in their jugular veins. Following catherization the ewes were placed in individual pens and kept there until the end of the infusion period. Then they were either ovariectomised immediately (the 2 luteal phase groups) or returned to group pens where they were left until ovariectomy 30 h after the end of infusion (the 2 follicular phase groups). During the experiment the ewes were fed a basal diet of good quality hay at maintenance levels [28] with *ad libitum* access to straw roughage and fresh water. The experiment was carried with local ethical approval and in accordance with French and European regulations on the care and welfare of animals in research and with ethical approval from the Ministry of Agriculture (N° 006259 and 2012-01-2).

**Figure 1:**
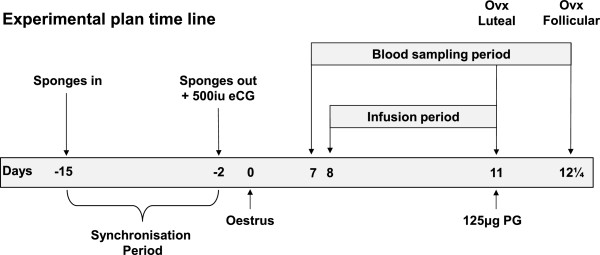
**The experimental plan.** The oestrous cycles of 36 ewes were synchronised using a combination of progestagen sponges and intramuscular eCG. On day 7 of the cycle following oestrous synchronisation, sampling of jugular venous blood commenced and continued until the time of ovariectomy. The next day (day 8) intravenous infusions of either saline (n = 18) or glucose (n = 18) commenced and continued for 72 hours. Ovariectomies were carried out at two times, the first at the end of the infusion period (luteal phase groups) and the second 30 h later and following the injection of a luteolytic dose on PGF given at the end of the infusion period (follicular phase groups).

The experimental plan is shown in Figure 1, briefly, oestrus was synchronised using progestagen sponges (Chronogest; Intervet/Schering-Plough Animal Health, Angers, France). Eight days after oestrus one group (n = 18) was infused with glucose at 10 mM/h for 72 h. A second group was infused with physiological saline at the same rate (n = 18) and acted as controls. At the end of the infusion ovaries were collected from half the ewes in each treatment group to form the two luteal phase groups. Luteolysis was induced with 125 μg of an analogue of PGF_2α_ (Cloprostenol; Intervet/Schering-Plough Animal Health, Angers, France) in the remaining ewes whose ovaries were collected 30 h after the end of infusion to form the two follicular phase groups. The body weights of the ewes were measured at the start of the infusion and at the time of ovariectomy.

### Blood collection

Two days before the start of the infusions both jugular veins were fitted with intravenous catheters. One catheter was used exclusively for infusions and the other exclusively for sampling blood. A sterile, 18% (w/v) solution of glucose was used and the rate of infusion was adjusted to deliver glucose at a rate of 10 mM per hour. The controls were infused with sterile saline at the same rate. The infusions were started on day 8 of the oestrous cycle and were continued for 72 h until day 11. Samples (5 mL) of jugular venous blood were collected regularly throughout the experiment as follow: For the determination of the plasma concentrations of oestradiol-17β, progesterone and FSH samples were taken every 6 h from -24 h relative to the start of infusion until ovariectomy. These samples were collected into lithium heparin tubes. For glucose and insulin samples were taken 24 hours before the start of the infusions and then at 0, 3, 9, 24, 48 and 72 hours after the start of the infusion. These samples were collected into fluoride/EDTA tubes. The blood samples were centrifuged at 4°C for 20 minutes at 1,000 g. The plasma was then decanted and stored at -20°C.

### Collection and dissection of ovaries

The animals were ovariectomised under pentothal-induced, halothane-maintained anaesthesia, by the laboratory veterinarian. Within a minute of removal, the ovaries were placed in ice-cold sterile saline for transport from the surgery to the laboratory. In the laboratory, the number of corpora lutea was noted, the ovaries were weighed and all follicles >1 mm in diameter were dissected out using fine scissors and fine toothed dissecting forceps. The isolated follicles were placed in ice-cold phosphate buffered saline (0.72 M Na_2_HPO_4_.2H_2_0; 0.28 M Na_2_H_2_PO_4_.2H_2_0 and 0.155 M NaCl at pH 7.0) in sterile, plastic Petri dishes and their average diameter measured to the nearest mm (for details, see below). From this point the follicles were processed individually as described [29]. Individual follicles were placed in a sterile plastic mini-Petri dish containing 1 ml of sterile phosphate buffered saline. Each follicle was then hemisected and the granulosa cell layer was gently scraped into the phosphate buffered saline using a fine plastic loop. The follicular shell of mainly theca cells was placed in a 1.5 mL micro-tube, snap frozen and stored at -80°C. The phosphate buffered saline containing, granulosa cells and follicular fluid was then placed into another 1.5 mL micro-tube and centrifuged at 4°C and 1,000 X g for 10 min. Following centrifugation, the supernatant containing diluted follicular fluid was transferred into a 1.5 mL micro-tube snap frozen and stored at -20°C. The separated granulosa cell pellet was also snap-frozen and stored at -80°C.

### Measurement and classification of follicles

The diameter of all dissected follicles was measured in two dimensions at 90°, using a graph paper grid placed below the Petri dishes that contained the dissected follicles and the average diameter calculated. The follicles were then grouped into three classes based on their diameters. These were: small >1.0-2.00 mm; medium >2.0 to 3.5 mm and large >3.5 mm. Follicles were also classified on the basis of their oestrogenicity. Oestrogenic follicles were defined as those with a concentration of oestradiol in follicular fluid >100 ng/mL, and non-oestrogenic follicles as those with <100 ng/mL. These definitions allow us to identify the following categories of follicles:

(i) Large (diameter >3.5 mm) oestrogenic follicles
(ii) Large non-oestrogenic follicles
(iii) Medium (diameter >2.0 to 3.5 mm) oestrogenic follicles
(iv) Medium non-oestrogenic follicles
(v) Small (diameter >1.0 to 2.0 mm) oestrogenic follicles.
(vi) Small non-oestrogenic follicles.

The volume of follicular fluid volume was calculated as described previously [30].

### Glucose and hormone assays

The jugular venous blood samples collected in fluoride EDTA tubes were analysed for glucose and insulin and those collected in heparin-lithium tubes were analysed for progesterone, oestradiol-17β and FSH. Samples of follicular fluid were assayed for oestradiol-17β. All assays were carried out in duplicate.

#### Glucose

The concentration of glucose in plasma was determined by colourimetry using the glucose oxidase method (Glucose Assay Kit [cat # G3660] and O-Dianisidine [cat # D2679] Sigma Aldrich Inc., Saint-Quentin Fallavier, France). The assay method followed the instructions provided by the manufacturer of the kit. Plasma samples were diluted in phosphate buffered saline (0.05 M, pH 7.6) as required, to obtain concentrations that fell within the range of the standard curve (2 to 8 mg of glucose per dL). The sensitivity of the assay was 2 mg/dL and the inter-assay and intra-assay coefficients of variation were 8% and 3% respectively.

#### Insulin

The concentration of insulin in plasma was measured using a heterologous radioimmunoassay developed in our laboratory [31]. The antiserum was rabbit anti-porcine insulin, the standards were ovine insulin and the second antibody was a goat anti-rabbit IgG (all reagents from Sigma Aldrich Inc., Saint-Quentin Fallavier, France). The sensitivity of the assay was 0.05 ng/mL and the inter-assay and intra-assay coefficients of variation were 10% and 15% respectively. The cross reactivity of the antiserum with ovine insulin was 100% relative to the homologous insulin standard.

#### Oestradiol-17β

The concentration of oestradiol-17β in plasma was determined by the radioimmunoassay of solvent extracted plasma [32] using a commercial oestradiol assay kit (Estradiol-2 kit P2210; Diasorin, SA, Antony, France). The limit of detection of oestradiol was 0.39 pg/mL and the inter- and intra-assay coefficients of variation were 20.5% and 6.0% respectively. The concentrations of oestradiol-17β in samples of follicular fluid were determined using the same commercial oestradiol radioimmunoassay. Samples of follicular fluid were first diluted 1:10 or 1:100 in phosphate buffered saline (0.05 M, pH 7.6) and assayed without solvent extraction. The concentration of oestradiol-17β in follicular fluid was derived using the theoretical volume of follicular fluid calculated as described [30], and corrected for dilution.

#### Follicle stimulating hormone (FSH)

The concentration of FSH in plasma was analysed using an ELISA [33]. The limit detection of FSH was 0.1 ng/mL the inter- and intra-assay coefficients of variation were 16.0% and 12.2% respectively.

#### Progesterone

The concentration of progesterone in plasma was determined using an ELISA [34]. The limit of detection of progesterone was 0.25 ng/mL the inter- and intra-assay coefficients of variation were 20.5% and 14.3% respectively.

### Antibodies for Western blotting

All antibodies were obtained from commercial sources and their details are presented in Table 1. The analysis of Akt used a rabbit polyclonal antibody to Akt (Cell Signalling Technology, Beverly, Ma., USA) and a rabbit polyclonal antibody to phospho-Akt1/2/3 (Ser473; Santa Cruz Biotechnology Inc., Heidelberg, Germany). The analysis of AMPKα1/2 used a rabbit polyclonal antibody to AMPK α1/2 (Cell Signalling Technology, Beverly Ma., USA) and a rabbit polyclonal antibody to phospho-AMPK (thr172; Cell Signalling Technology, Beverly, Ma., USA). The analysis of ERK2 used a polyclonal antibody to ERK2 (Cell Signalling Technology, Beverly, Ma., USA) and a polyclonal antibody to phospho-ERK1/2 (Thr202/Tyr204; Cell Signalling Technology, Beverly, Ma., USA). A mouse monoclonal antibody was used to analyse Aromatase P_450_ (ABD Serotec, Düsseldorf, Germany) and a mouse monoclonal antibody to vinculin was used as an internal standard (Sigma Aldrich Inc., Saint-Quentin Fallavier, France). All antibodies were used at a dilution of 1/1000 dilution. The secondary antibodies that were used depending on the species used to generate the primary antibody were either a goat anti-mouse IgG (Laboratories Eurobio, Courtaboeuf, France) or a goat anti-rabbit IgG (Laboratories Eurobio, Courtaboeuf, France).

**Table 1:** **Details of the primary and secondary antibodies used for western immuno-blotting in this experiment**

Antibody	Type of antibody	Species	Source	Final dilution
***Primary***	Anti-cytochrome _P450_ Aromatase _P450_	Mouse monoclonal	ABD Serotec, Cergy Pontoise, France	1:200
	Anti-Akt	Rabbit polyclonal	Cell Signalling (OZYME), Saint Quentin Yvelines, France	1:1,000
	Anti-phospho-Akt1/2/3 (Ser473)-R	Rabbit polyclonal	Santa Cruz Biotechnology, Tebu-Bio, Le Perray-en_ Yvelines	1:1,000
	Anti-AMPK	Rabbit polyclonal	Cell Signalling (OZYME), Saint Quentin Yvelines, France	1/1,000
	Anti-phospho-AMPK Thr 172	Rabbit polyclonal	Cell Signalling (OZYME), Saint Quentin Yvelines, France	1:1,000
	Anti-Erk2 (C-14)	Rabbit polyclonal	Cell Signalling (OZYME), Saint Quentin Yvelines, France	1:1,000
	Anti-phospho-ERK1/2	Rabbit polyclonal	Cell Signalling (OZYME), Saint Quentin Yvelines, France	1/1,000
	Anti-vinculin	Mouse monoclonal	Sigma Aldrich, Saint-Quentin Fallavier, France	1:1,000
***Secondary***	Anti-Mouse IgG	Goat polyclonal	Laboratoires Eurobio, Courtaboeuf, France	1:10,000
	Anti-Rabbit IgG	Goat polyclonal	Laboratoires Eurobio, Courtaboeuf, France	1:10,000

### Western blotting

Western blotting was used to determine the levels of Aromatase P_450_ protein relative to vinculin (an internal standard) in granulosa cell lysates from individual follicles greater than 2 mm in diameter [18], Aromatase P_450_ was not determined in small follicles. The levels of phosphorylated Akt and total Akt [18] were determined in granulosa cell lysates from individual follicles greater than 2 mm in diameter. The granulosa cells harvested from follicles between 1 and 2 mm in diameter were pooled within sheep. Similarly, the levels of phosphorylated AMPK and total AMPK [18] and the levels of phosphorylated ERK1/2 and total ERK2 were determined in granulosa cell lysates from individual follicles greater than 2 mm in diameter and in granulosa cells pooled within ewes from follicles between 1 and 2 mm in diameter. Lysates of granulosa cells were prepared as described [18] and then analysed by western blotting. The concentration of protein in the supernatants was determined by colourimetry using the BCA protein assay reagent (Interchim, Montluçon France). Aliquots of lysate containing 30 μg of protein were re-suspended in Laemmli buffer (glycerol 50%, SDS 10%, Hepes 1 M-pH7.6, beta-Mercaptoethanol 25%, bromophenol blue) and then analysed by western blotting.

Granulosa cell lysates were subjected to electrophoresis on 10% (v/v) SDS-polyacrylamide gels for 2.5 h at 80 V, in the running buffer (H_2_0_2_, 50 mM Tris Base, 400 mM Glycine, 2% EDTA 0.1 M, 1% SDS 10%). The proteins were then transferred onto nitrocellulose membranes for 1.5 h at 80 V, in transfer buffer (H_2_0_2_, 20 mM Tris Base, 200 mM Glycine, 20% Methanol, 0.1% SDS 10%). After washing in TBS Tween (H_2_0_2_, 2 mM Tris Base, 15 mM NaCl, 0.1% Tween 20, pH 7.4), the membranes were incubated for 1 h at room temperature with TBS Tween containing 5% dry milk powder to saturate non-specific sites. Subsequently, membranes were incubated overnight at 4°C with primary antibodies in TBS Tween containing 5% dry milk powder.

After washing in TBS Tween, the membranes were incubated with a horseradish peroxidase (HRP)-conjugated anti-rabbit IgG (Final dilution 1:10 000; Laboratories Eurobio, Courtaboeuf, France) or horseradish peroxidase (HRP)-conjugated anti-mouse IgG (Final dilution 1:10,000; Laboratories Eurobio, Courtaboeuf, France) for 2 h at room temperature in TBS Tween containing 5% dry milk powder. After washing in TBS Tween, the signal was detected by enhanced chemiluminescence (PerkinElmer, Life and Analytical Sciences, Courtaboeuf, France). The membranes were exposed on GE Healthcare film (PerkinElmer, Life and Analytical Sciences, Courtaboeuf, France), and then developed (Kodak AL4) and fixed (Kodak LX24) and dried. The films were analysed and the blots quantified using ScionImage (4.0.3.2 version, Scion Corporation, Frederick, Maryland, USA).

### Statistical analysis

Statistical analyses were performed using specialised software for statistical analysis (SAS Statview version 5.0). Data on hormone concentrations in plasma and body weight were analysed by repeated measures ANOVA with time as the repeated measure. Where it was appropriate, *post-hoc* paired comparisons within time were carried out using the Bonferroni test. Other data were analysed by univariate ANOVA apart from the data on follicle number which were analysed using the Chi-squared test. The data are presented as the mean ± sem and differences are regarded as significant when P < 0.05.

## Results

In a ewe from a glucose-infused group the catheter failed and the infusion was interrupted. This animal was excluded from the experiment.

### Body weight

The average weights in the group infused with glucose at the start of the infusion and at ovariectomy were 47.0 ± 0.72 and 45.6 ± 0.78 kg, respectively. The average weights in the control group at the start of infusion, and at ovariectomy were 48.0 ± 0.73 and 46.0 ± 0.84 kg, respectively. There were no significant differences in the average weights within or between groups.

### Ovarian morphology

The ewes in the two luteal phase groups had 3.11 ± 0.80 functional corpora lutea (CLs) in the glucose-infused group and 2.89 ± 0.58 CLs in the control group, confirming that the ewes were as expected, in the luteal phase of the oestrous cycle. The numbers of follicles dissected from the ovaries of control and glucose infused ewes are shown in Table 1. There were significantly more small follicles in the glucose-infused groups compared to controls at the same stage of the oestrous cycle (Table 2) and this is reflected also in the total number of follicles. There were no differences in follicle numbers between stages of the oestrous cycle and the number of medium and large follicles was not different between glucose-infused and control ewes (Table 2).

**Table 2:** **Corpora lutea (CLs) and follicles**

Phase	Treatment	CLs	Follicles			
			Small	Medium	Large	Total
***Glucose (n = 17)***	Follicular (n = 8)	2.4 ± 0.32	26.0 ± 1.92^a^	7.9 ± 0.78	2.0 ± 0.53	36.9 ± 3.57^a^
	Luteal (n = 9)	3.1 ± 0.8	27.2 ± 3.65^a^	10.8 ± 1.52	1.9 ± 0.42	39.9 ± 4.43^a^
***Saline (n = 18)***	Follicular (n = 9)	1.8 ± 0.32	16.2 ± 0.99^b^	6.9 ± 1.85	2.1 ± 0.48	25.5 ± 1.49^b^
	Luteal (n = 9)	2.9 ± 0.58	13.1 ± 1.67^b^	10.1 ± 1.29	1.9 ± 0.45	25.1 ± 2.78^b^

The mean ± SEM, number of corpora lutea (CLs) and follicles classified by diameter as small (1 to <2 mm) medium (2 to <3.5 mm) and large (>3.5 mm) of ewes during the follicular and luteal phases of the oestrous cycle and following a 72 h infusion of saline or glucose (10 mM/h). Within columns, values with different superscripts (a, b) are significantly different at P < 0.05.

### Plasma concentrations of metabolites

#### Glucose

**Figure 2:**
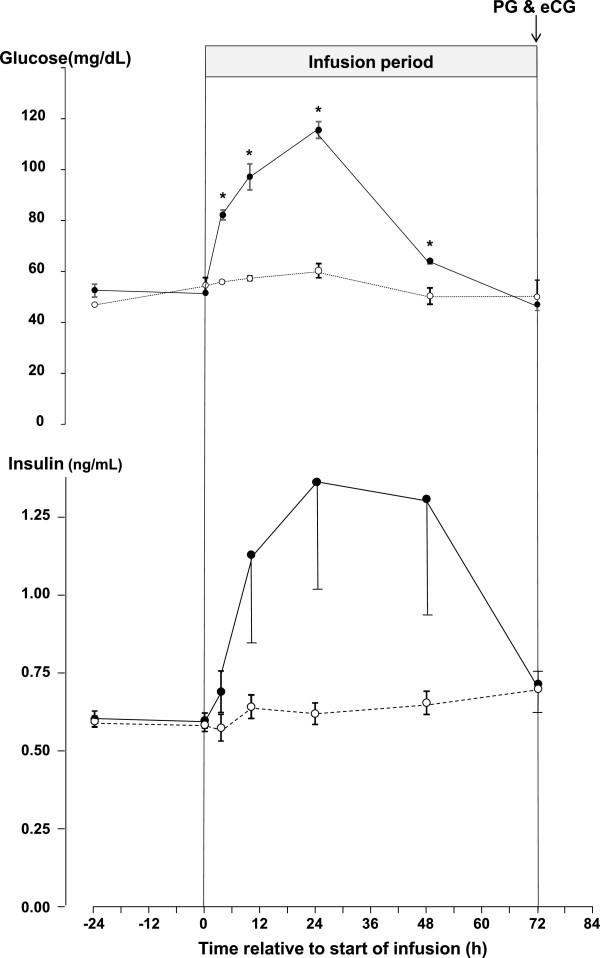
**The mean ± sem, concentrations of glucose (upper graph) and insulin (lower graph) in jugular venous plasma of ewes infused with either saline (n = 18; dashed line with open circles) or with 10 mM/h of glucose (n = 17; solid lines with closed circles) for 72 h during the late luteal phase of the oestrous cycle.** Within times, an asterisk (*) indicates a significant difference (P < 0.05).

The concentrations of glucose in plasma are shown in Figure 2, overall there was a significant effect (P = 0.001) of treatment on the concentration of glucose. The plasma concentration of glucose was significantly elevated by 3 h (P <0.01) after the start of the infusion of glucose and remained elevated at 9 h (P <0.01), at 24 h (P <0.01) and at 48 h (P <0.05) but, it had decreased to control concentrations at 72 h. The concentration of plasma glucose in control ewes did not vary significantly during the experiment (Figure 2).

#### Insulin

The plasma concentrations of insulin are shown in Figure 2, overall there was a significant effect (P = 0.02) of treatment on the concentration of insulin. Paired comparisons revealed that the concentration of insulin was significantly elevated by 9 h (P <0.05) after the start of the infusion of glucose and remained elevated at 24 h (P <0.01) and at 48 h (P <0.05) but, it had decreased to control concentrations at 72 h. The concentration of insulin in control ewes did not vary significantly during the experiment (Figure 2).

### Plasma concentrations of reproductive hormones

#### Oestradiol-17β

**Figure 3:**
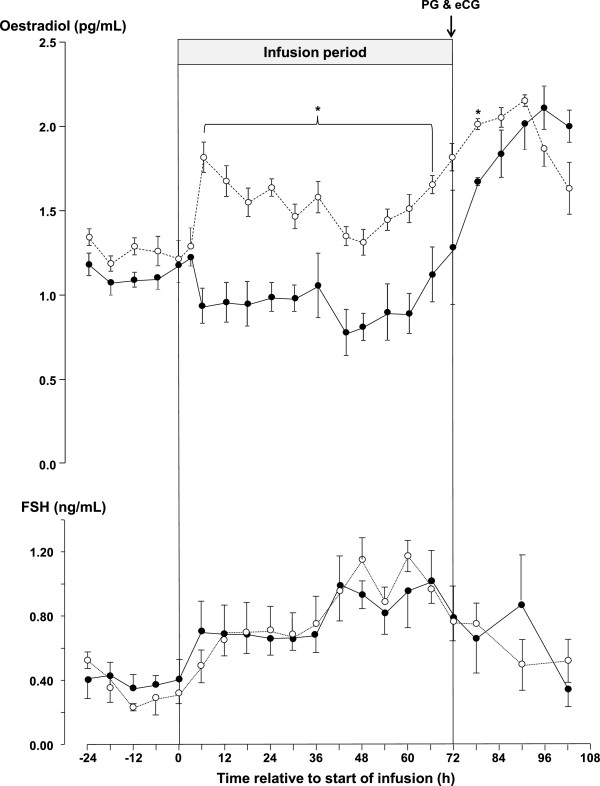
**The mean ± sem, concentrations of oestradiol-17β (upper graph) and FSH (lower graph) in jugular venous plasma of ewes infused with either saline (n = 18 and n = 9 after 72 h; dashed line with open circles) or with 10 mM/h of glucose (n = 17 and n = 8 after 72 h; solid lines with closed circles) for 72 h during the late luteal phase of the oestrous cycle.** Within times, an asterisk (*) indicates a significant difference (P < 0.05).

The plasma concentrations of oestradiol-17β are shown in Figure 3. The pre-treatment concentrations of plasma oestradiol were not significantly different between the glucose and control groups. However, the plasma concentration of oestradiol was reduced in the glucose-infused groups compared to the control group (P <0.05) during the infusion period between 6 and 66 h after the start of the infusions. There was also a significant effect of time (P <0.05) following luteolysis; the plasma concentration of oestradiol increased significantly in both groups and by 12 h after PG the glucose-infused group was no longer lower than the control group (Figure 3).

#### Follicle stimulating hormone (FSH)

The concentration of FSH in plasma (0.72 ± 0.040 ng/mL) from glucose-infused ewes was not different from that in control ewes (0.67 ± 0.036 ng/mL). There was a significant effect of time and the concentration of FSH fell significantly following the induction of luteolysis (Figure 3) but, the interaction between time and treatment was not significant.

#### Progesterone

The profiles of progesterone in plasma (all concentrations above 2 ng/mL prior to the injection of PG and falling to below 1 ng/mL by 30 h after PG) confirmed that all ewes were undergoing normal oestrous cycles and that they were at the correct stage of the oestrous cycle at the time the ovaries were collected.

#### Concentrations of oestradiol in follicular fluid

**Figure 4:**
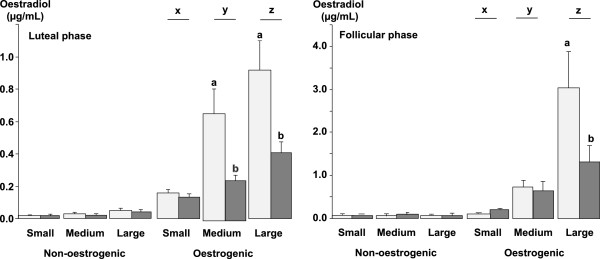
**The mean ± sem, concentrations of oestradiol-17β in follicular fluid from small (<2.0 mm), medium (2.0 to 3.5 mm) and large (>3.5 mm) diameter oestrogenic (oestradiol >100 ng/mL) and non-oestrogenic (oestradiol <100 ng/mL) follicles from ewes during the luteal and follicular phases of the oestrous cycle infused with saline (light grey columns) or with 10 mM/h of glucose (dark grey columns) for 72 h during the late luteal phase of the oestrous cycle.** Follicle classes with different letters (x, y and z) differ significantly at P < 0.05. Note there is a difference in scale between the luteal and follicular phase diagrams.

The concentrations of oestradiol in follicular fluid are shown in Figure 4. The concentration of oestradiol in oestrogenic follicles was significantly higher compared to non-oestrogenic follicles from all three follicle classes (P < 0.05) and in the follicular phase compared to the luteal phase of the oestrous cycle (P < 0.05; Figure 4). The concentration of oestradiol did not differ significantly among follicle classes in non-oestrogenic follicles but, in oestrogenic follicles the concentration of oestradiol was higher in medium follicles compared to small follicles (P < 0.001) and higher in large follicles compared to medium (P < 0.001) and small follicles (P < 0.001; Figure 4). Finally the infusion of glucose reduced the concentration of oestradiol in large (P < 0.001) oestrogenic follicles collected during the follicular phase of the oestrous cycle and in both medium (P < 0.001) and large (P < 0.001) oestrogenic follicles collected in the luteal phase of the oestrous cycle (Figure 4). Glucose had no effect in non-oestrogenic follicles (Figure 4).

### Aromatase P_450_

**Figure 5:**
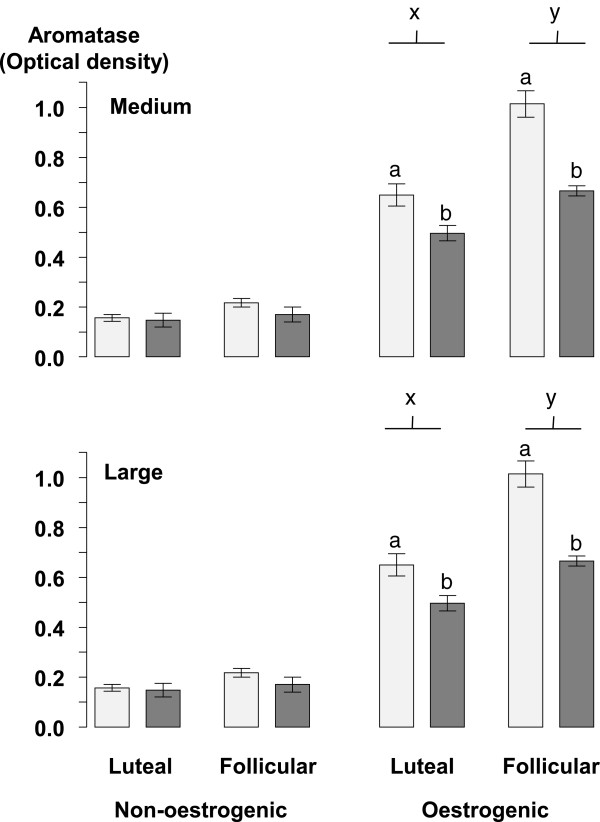
**The mean ± sem, level of aromatase P**_**450**_ **protein in granulosa cell lysates from medium (2.0 to 3.5 mm) and large (>3.5 mm) diameter oestrogenic (oestradiol >100 ng/mL) and non-oestrogenic (oestradiol <100 ng/mL) follicles from ewes during the luteal and follicular phases of the oestrous cycle infused with saline (light grey columns) or with 10 mM/h of glucose (dark grey columns) for 72 h during the late luteal phase of the oestrous cycle.** Follicle classes with different letters (x and y) and columns with different letters (a and b) differ significantly at P < 0.05.

The levels of Aromatase P_450_ protein in granulosa cells are shown in Figure 5. In oestrogenic follicles Aromatase P_450_ was significantly (P < 0.05) higher in the follicular phase compared to the luteal phase in both medium and large follicles. The levels of Aromatase P_450_ were significantly (P < 0.05) lower in non-oestrogenic follicles compared to oestrogenic follicles regardless of their size (Figure 5). The infusion of glucose significantly (P < 0.05) reduced the level of Aromatase P_450_ in oestrogenic follicles of all sizes but, it had no effect on the level of Aromatase P_450_ in non-oestrogenic follicles (Figure 5). In non-oestrogenic follicles Aromatase P_450_ did not vary significantly with either the stage of the oestrous cycle or the diameter of the follicle.

### Mediators of Insulin-signalling (Akt, ERK and AMPK)

**Figure 6:**
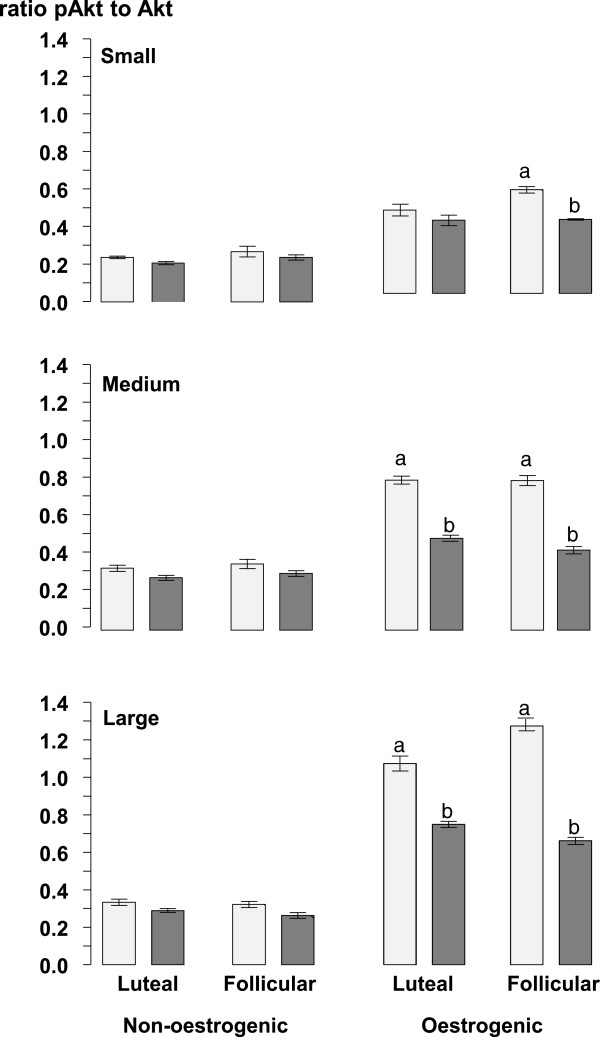
**The mean ± sem, ratios of phosphorylated to non-phosphorylated Akt in granulosa cell lysates from small (<2.0 mm), medium (2.0 to 3.5 mm) and large (>3.5 mm) diameter oestrogenic (oestradiol >100 ng/mL) and non-oestrogenic (oestradiol <100 ng/mL) follicles from ewes during the luteal and follicular phases of the oestrous cycle infused with saline (light grey columns) or with 10 mM/h of glucose (dark grey columns) for 72 h during the late luteal phase of the oestrous cycle.** Columns with different letters (a and b) differ significantly at P < 0.05.

**Figure 7:**
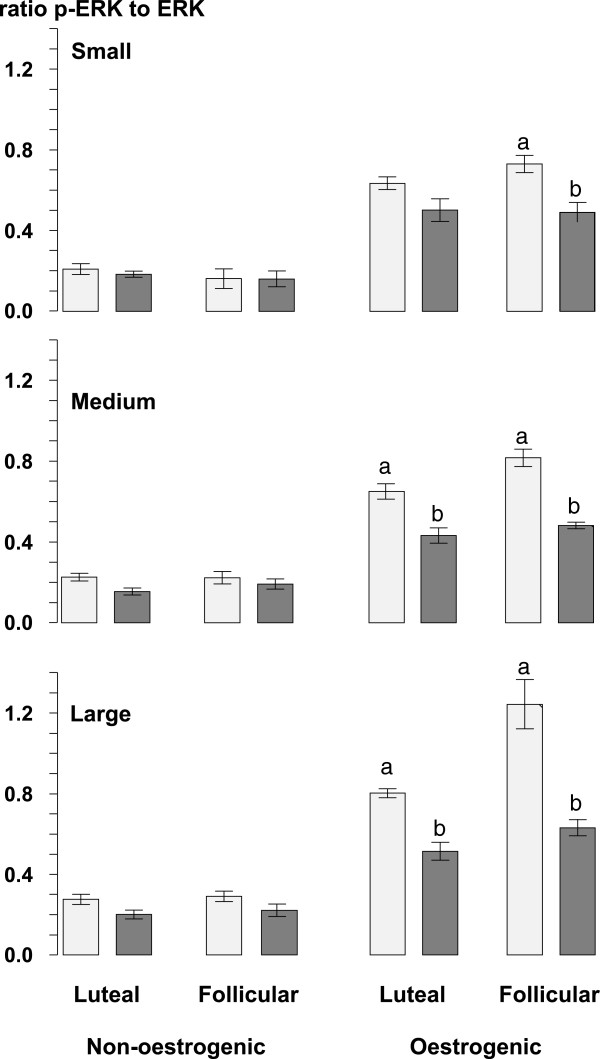
**The mean ± sem, ratios of phosphorylated to non-phosphorylated ERK in granulosa cell lysates from small (<2.0 mm), medium (2.0 to 3.5 mm) and large (>3.5 mm) diameter oestrogenic (oestradiol >100 ng/mL) and non-oestrogenic (oestradiol <100 ng/mL) follicles from ewes during the luteal and follicular phases of the oestrous cycle infused with saline (light grey columns) or with 10 mM/h of glucose (dark grey columns) for 72 h during the late luteal phase of the oestrous cycle.** Columns with different letters (a and b) differ significantly at P < 0.05.

**Figure 8:**
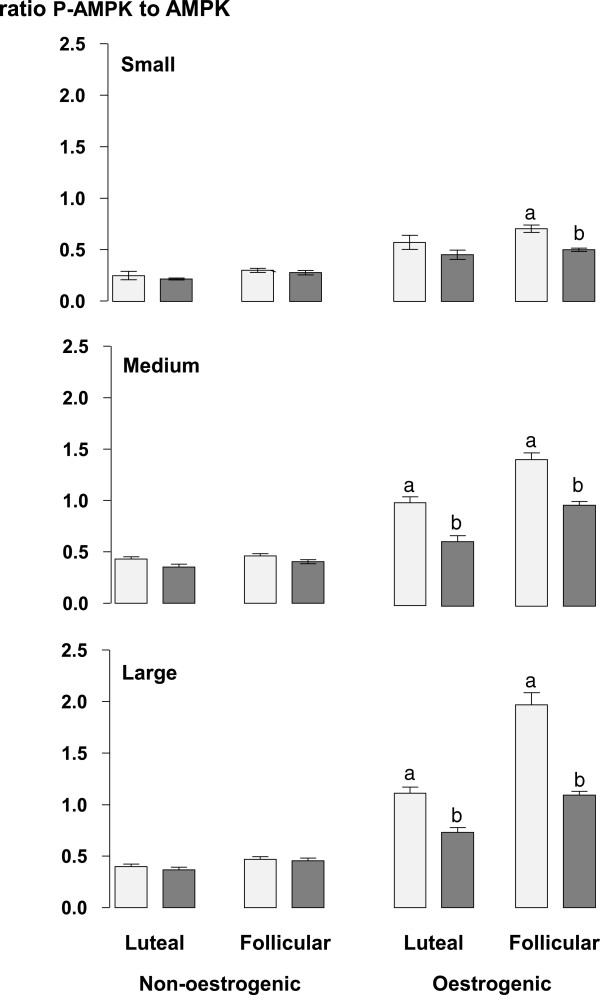
**The mean ± sem, ratios of phosphorylated to non-phosphorylated AMPK in granulosa cell lysates from small (<2.0 mm), medium (2.0 to 3.5 mm) and large (>3.5 mm) diameter oestrogenic (oestradiol >100 ng/mL) and non-oestrogenic (oestradiol <100 ng/mL) follicles from ewes during the luteal and follicular phases of the oestrous cycle infused with saline (light grey columns) or with 10 mM/h of glucose (dark grey columns) for 72 h during the late luteal phase of the oestrous cycle.** Columns with different letters (a and b) differ significantly at P < 0.05.

The ratios of phosphorylated to non-phosphorylated forms of Akt (Figure 6), ERK (Figure 7) and AMPK (Figure 8) were all lower (all at P <0.05) in non-oestrogenic follicles compared to oestrogenic follicles while in non-oestrogenic follicles the ratios for all three were unaffected by the diameter of the follicle, the stage of the oestrous cycle or the infusion of glucose (Figures 6, 7 and 8). However, in oestrogenic follicles the infusion of glucose reduced the ratio of phosphorylated non-phosphorylated forms of all three compared to control ewes in medium and large follicles from the luteal phase (all at P <0.05) and in all three follicle classes in follicles from the follicular phase (P <0.05; Figures 6, 7 and 8). Similarly, the ratios for all three were not significantly different in small and medium follicles and they were significantly higher in large follicles (all at P <0.05) compared to small and medium follicles (Figures 6, 7 and 8). There were no significant differences between the follicular and luteal phases between follicle types or among follicle diameters.

## Discussion

The results of this experiment show that in cyclic ewes during the breeding season, the intravenous infusion of glucose increased the total number of follicles present in their ovaries [17]. These findings agree with those obtained when using anoestrous ewes treated with eCG [18]. Furthermore the present experiment also shows that the effect of intravenous glucose on the follicle population of the ovary is present in both the luteal and follicular phases of the oestrous cycle. The concentrations of glucose achieved by the infusions peaked at 120 mg/dL or about 30% above the upper limit of the normal reference range for sheep (45-80 mg/dL). Additionally, this effect of glucose was also associated with a reduction in the circulating concentrations of oestradiol (Figure 3) but without any associated change in the concentrations of circulating FSH (Figure 3) and a reduction in the level of ovarian Aromatase P_450_ in oestrogenic follicles (Figure 5). Furthermore, the effect of glucose was accompanied by an increase in the circulating concentration of insulin (Figure 2) and finally it was associated with changes in the insulin signalling and energy sensing mechanisms (Figures 6, 7 and 8) in granulosa cells.

Very similar findings have been reported for the effects of diets that increase the supply of energy. Feeding dietary supplements such as lupin grain [13, 17, 20, 35, 36], a mixture of soya meal and maize [14], or steamed corn flakes [37] or administering by gavage, an energy supplement in the form of a mixture of glycerol and propylene glycol [38, 39] to ewes all increased the number of follicles in their ovaries. In all these studies the nutritional treatment increased the concentrations of glucose and insulin in jugular venous plasma and these changes were associated with an increased number of follicles. Some of these studies also reported reduced concentrations of oestradiol in jugular plasma [20, 35], reduced levels of Aromatase P_450_ in granulosa cell lysates [13, 20] and lower concentrations of oestradiol in follicular fluid from oestrogenic follicles [13, 20].

There is some controversy over the effects of either glucose or glucogenic diets on the circulating concentrations of oestradiol. We have reported decreased circulating concentrations of oestradiol following the infusion of glucose [18] or the feeding of a lupin grain supplement [20] and other authors have reported similar findings [35]. However other studies from our group found that feeding lupin grain [13] had no effect on oestradiol and other authors have also found no effect of glucogenic diets on the concentrations of oestradiol in jugular venous blood [14]. Using a different experimental model, these authors also reported that a supplement of lupin grain increased oestradiol concentrations and reduced those of FSH [36]. The main reason for these discrepancies is most probably because sheep have particularly low concentrations of oestradiol [40] and the oestradiol assays employed are being used at the very limits of their technical capability. The secretion rate of oestradiol was reduced by feeding a lupin grain supplement to ewes with ovarian auto-transplants [3] and in other experiments the concentration of oestradiol in the follicular fluid was reduced by feeding a supplement of lupin grain [13, 20] or a high energy diet [41] and in this experiment the infusion of glucose reduced the concentration of oestradiol in the follicular fluid of oestrogenic follicles (Figure 4). Furthermore these treatments were associated with reduced levels of Aromatase P_450_ in granulosa cell lysates from oestrogenic follicles [13, 18, 20, 42]. So on balance we conclude tentatively, that it is very likely that both intravenous glucose and glucogenic diets can reduce the secretion of follicular oestradiol from oestrogenic follicles.

The effect of glucose on ovarian follicles appears to be dependent on the stage of development of the follicles. In the population of small follicles the effect of glucose was to stimulate their growth a conclusion that can be inferred from the increased number of small follicles in glucose-infused ewes (Table 2). Furthermore in small non-oestrogenic follicles this effect was not associated with any detectable change in the level of phosphorylated Akt, ERK1/2 and AMPK (Figures 6, 7 and 8) suggesting that this effect of glucose was not related to altered activity of insulin-signalling pathways in granulosa cells. This leads us to conclude that it is was possibly a direct effect of an increased supply of metabolic fuel (glucose) to the ovary. By contrast, in medium and large follicles glucose had little effect on the number of follicles (Table 2) but, in medium and large oestrogenic follicles it did have substantial effects on their physiological function through an inhibition of the level of aromatase P_450_ (Figure 5) and a reduction in the concentration of oestradiol in follicular fluid (Figure 4). These actions were associated with changes in insulin signalling pathways and so these follicles are presumed to be responsive to both insulin and glucose and that the effect of glucose is insulin-mediated [43].

Extrapolating further, these data indicate that oestrogenic follicles are insulin-responsive [43] and that non-oestrogenic follicles are not. The population of non-oestrogenic follicles is a mixed population consisting of a sub-population of undifferentiated, small growing follicles and a sub-population of medium and large atretic follicles neither of which are insulin-responsive. Our findings suggest that glucose acts directly in growing follicles to stimulate growth leading to greater numbers of small follicles while in oestrogenic follicles which are physiologically functional but non-growing, glucose acts indirectly through an insulin-mediated mechanism to inhibit the synthesis and secretion of oestradiol [43].

The circulating concentration of IGF-I was not determined in this experiment. However, it was in our earlier experiment [18] where glucose increased the jugular venous concentrations of IGF-I. Despite the fact that IGF-I is a potent stimulator of both follicle growth [29] and oestradiol secretion [29, 44] we consider it unlikely that the effects of glucose in the follicle, in this experiment were mediated by IGF-I. IGF-I is a potent growth factor controlled by hepatic feedback systems that are independent of ovarian follicles [45]. Consequently there are intra-ovartian mechanisms that regulate the activity of IGF-I [46, 47] to protect the follicle from the potentially harmful effects of high concentrations of IGF-I. None-the-less it is possible that the insulin-independent effects of glucose on small follicles (Table 2) are an effect of IGF-I. Interestingly, glucose reduced circulating (Figure 3) and intrafollicular (Figure 4) concentrations of oestradiol suggesting that the intra-follicular bio-activity of IGF-I in oestrogenic follicles is suppressed.

The expected effect of reduced secretion of oestradiol (Figure 3) would be an increase in circulating FSH. How then can we explain the absence of the expected compensatory increase in FSH? Published data on the effects of glucogenic diets on the plasma concentrations of FSH presents a mixed picture. Some authors have reported increased concentrations of FSH and others have reported no change. It has been suggested that the reasons for these differences are technical and associated with the assays employed to measure ovine FSH [45] or that they reflect the dynamic nature of the negative relationship between FSH and the follicle [3]. However, it is also possible that the variation simply indicates the presence of uncontrolled and unidentified physiological differences among animals leading to unconscious bias in randomisation procedures. In an attempt to control this variability, Vinoles and her colleagues [36] used a first wave model to synchronise follicles waves and thus theoretically, reduce between ewe variability in the follicle population. Their results showed that supplementation with a glucogenic diet increased concentrations of oestradiol and reduced those of FSH in jugular plasma. However, the control of FSH by negative feedback from the ovary has two hormonal components namely oestradiol and inhibin and until the effects of glucogenic diets on the secretion of ovarian inhibin have been described a complete understanding of the effects of glucogenic diets on FSH will not be possible. Regrettably, at present there in no satisfactory technique available for measuring the concentration of inhibin in jugular venous plasma from sheep.

It is worth noting that the population of small growing follicles is an important source of inhibin [48]. Because glucogenic diets increase their number then theoretically, these diets should also increase the total secretion of follicular inhibin and the concentration of inhibin in the peripheral circulation. We can speculate that the resulting increase in inhibin negative feedback on FSH may thus compensate for any reduced oestradiol feedback and may explain why in some studies oestradiol decreased but FSH did not increase. We suggest that variability of the FSH responses to glucogenic treatments may reflect subtle changes in the balance of the effects of glucogenic diets on small growing (i.e. inhibin secreting) and large oestrogenic (i.e. oestradiol secreting) follicles.

The follicle has a functional insulin-glucose system [5] and the level of activity in the pathway can be estimated from the ratio of phosphorylated to non-phosphorylated forms of the various protein kinases in the pathway. Some of the kinases activated by insulin, although not exclusively, including Akt, ERK and AMPK have been detected in ovarian follicles from ewes [18, 49–52] and in this experiment the phosphorylation ratios of all three of these were altered by the infusion of glucose. The Irish group [49–51] have shown that both Akt and ERK are implicated in the mechanism of follicle selection and this study confirms their findings [18] showing that intravenous glucose decreased the level of phosphorylation of Akt and AMPK and furthermore, shows for the first time that glucose reduced the level of phosphorylation of ERK. How do these effects relate to the mechanism through which a glucogenic diet can increase ovulation rate? Ovulation rate in sheep is ultimately determined by the number of gonadotrophin-dependent, ovulatory follicles present at the time of the LH surge. The reduction in the secretion of oestradiol by the ovulatory follicle suggests that the effect of glucose is to impair FSH-stimulated synthesis of oestradiol, the reduced levels of Aromatase P_450_ are consistent with such an action, and reduce its inhibitory influence (or dominance) on subordinate follicles in the cohort thus allowing for the emergence of additional ovulatory follicles. Potential mechanisms probably involve “cross-talk” [53] between the insulin signalling and FSH signalling pathways in granulosa and theca cells of the ovulatory follicle [5]. However, the details of how and where these signalling pathways interact are topics for future research.

## Conclusions

These results show that an intravenous glucose that maintains the concentration of blood glucose slightly above the upper end of the normal reference range increased the number of small follicles and altered the function of follicular granulosa cells in large oestrogenic follicles but not in small follicles or non-oestrogenic follicles of any size. The glucose reduced the levels of Aromatase P_450_ in large oestrogenic follicles and this was reflected in a reduced blood concentration of oestradiol. These reduced levels of Aromatase P_450_ were associated with reduced levels of phosphorylated Akt, ERK and AMPK in large follicles. However, glucose did not affect the phosphorylation state of the energy sensor, AMPK and mediators of insulin action (Akt, ERK) in small follicles. These data suggest that the effect of glucose in small follicles is a direct action of glucose that increases the number of small follicles while the effect of glucose in large, oestrogenic follicles is an indirect insulin-mediated action that inhibits their capacity to secrete oestradiol.

## Abbreviations

Akt: A Protein kinase B
AMPK: 5' adenosine monophosphate-activated kinase
ANOVA: Analysis of variance
BCA: Bicinchoninic acid
CL: Corpus luteum
eCG: equine chorionic gonadotrophin
EDTA: Ethylenediaminetetra acetic acid
ELISA: Enzyme linked immunosorbent assay
ERK: Extra cellular-related protein kinase
FSH: Follicle stimulating hormone
GH: Growth hormone
GLUT 4: Glucose transporter 4
IGF-I: Insulin-like growth factor 1
IgG: Immunoglobulin G
INRA: L’Institut National de la Recherche Agronomique
IRS: Insulin receptor substrate
LH: Luteinizing hormone
PGF_2α_: Prostaglandin F_2α_
SDS: Sodium dodecyl sulphate
Sem: Standard error of the mean
SLC2A-4: Sugar transport facilitator
TBS: Tris-buffered saline.
